# Local delivery of a novel PTHrP *via* mesoporous bioactive glass scaffolds to improve bone regeneration in a rat posterolateral spinal fusion model

**DOI:** 10.1039/c8ra00870a

**Published:** 2018-04-03

**Authors:** Bo Liang, Jinghuan Huang, Jianguang Xu, Xiaolin Li, Jingfeng Li

**Affiliations:** Department of Orthopedic Surgery, Shanghai Jiao Tong University Affiliated Sixth People's Hospital 600 Yishan Road Shanghai 200233 PR China jianguangxu2004@aliyun.com lixiaolin@sjtu.edu.cn; Department of Orthopedics, Zhongnan Hospital of Wuhan University Wuhan 430071 PR China Jingfengli@whu.edu.cn

## Abstract

With the development of tissue engineering, bone defects, such as fractured long bones or cavitary lesions, may be efficiently repaired and reconstructed using bone substitutes. However, high rates of fusion failure remain unavoidable in spinal fusion surgery owing to the lack of appropriate materials for bone regeneration under such challenging conditions. Parathyroid hormone (PTH), a major regulator of bone remodeling, exerts both anabolic and catabolic effects. In this study, we modified PTH(1–34) and designed and synthesized a novel PTH-related peptide, namely PTHrP-1. Further, we fabricated a local PTHrP delivery device from mesoporous bioactive glass (MBG) to address the need for a suitable material in spinal fusion surgery. Using MBG scaffolds as a control, the biological properties of PTHrP-MBG scaffolds were evaluated in terms of attachment, proliferation, and alkaline phosphatase activity, as well as osteogenic gene and angiogenic gene expression in co-cultured rat bone marrow mesenchymal stem cells (rBMSCs) *in vitro*. Furthermore, PTHrP-1-MBG scaffolds were tested in a rat posterolateral spinal fusion model. Our data showed that PTHrP-1-MBG scaffolds possessed good ability to facilitate attachment and stimulation of rBMSC proliferation and differentiation. Importantly, the *in vivo* results revealed that the PTHrP-1-MBG scaffolds facilitated faster new bone formation and a higher rate and quality of spinal fusion. Therefore, the results suggest that devices consisting of the present novel PTHrP and MBG possess wider potential applications in bone regeneration and should serve as a promising bone substitute for spinal fusion.

## Abstract 2

With the development of tissue engineering, bone defects, such as fractured long bones or cavitary lesions, may be efficiently repaired and reconstructed using bone substitutes.

## Graphical abstract

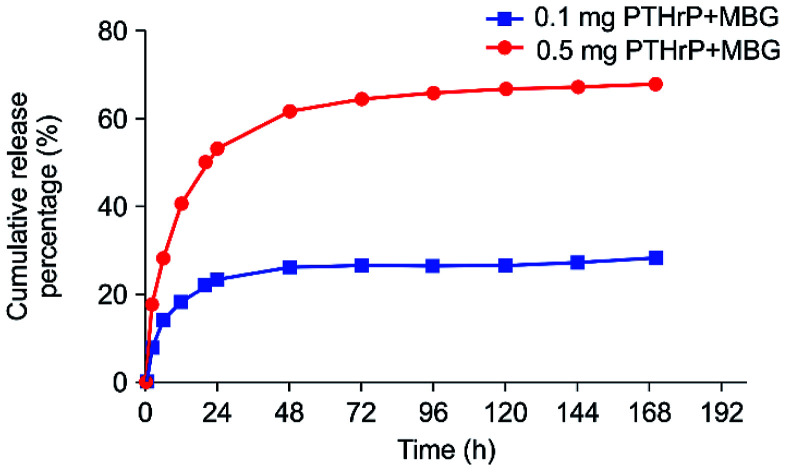

## 1. Introduction

With an increasingly aging population, the prevalence of clinical conditions affecting the spinal column that require surgical treatment is rising. For the purpose of facilitating patient comfort and mobility, spinal fusion surgery has significantly increased over the last few decades.^1,2^ To achieve and maintain fusion and intersegmental stability, bone grafts and bone substitutes are frequently adopted.^1^ Autologous bone was recognized as the gold standard for obtaining satisfactory spinal fusion.^2^ However, in addition to the limited supply of autologous bone graft, donor site morbidity, and poor bone quality in osteoporotic patients limit the effectiveness of this option.^3,4^ Although allograft bone is widely available, the problems of resorption, exposure, and disease transmission have restricted its use.^5^ In order to resolve this challenge, researchers in the orthopedic community have attempted to identify effective alternative methods. With the development of bone substitutes, a co-delivery system consisting of osteoinductive scaffolding material, growth factors, and osteogenic cells has been considered the most efficient procedure. Among various growth factors, BMP, which has achieved the highest radiographic fusion rates, has attracted the most attention.^1^ However, the elevated risk of wound-related complications and other complications, such as soft tissue swellings, uncontrolled heterotopic ossification, and radiculitis, limit the wider therapeutic administration of these osteoinductive molecules.^6–8^ Existing biomaterials fail to provide an efficacious and safe solution for bone regeneration without concomitant complications, and tissue engineering represents a novel therapy for reliable and efficient bone regeneration.

Parathyroid hormone (PTH) is an 84-amino-acid polypeptide secreted from the parathyroid gland, that is essential for the maintenance of calcium homeostasis through, in part, its regulation of bone remodeling.^9,10^ Teriparatide (PTH 1–34), the N-terminal fragment of the intact PTH, which is approved for use in the treatment of osteoporosis, accelerates bone union after instrumented lumbar posterolateral fusion.^11,12^ While teriparatide stimulates both bone resorption and bone formation, the final effect on bone mass, either catabolic or anabolic, depends on the dose and periodicity at which the hormone was administered.^13^ It has been shown by many investigators that intermittent or short-term, high-dose teriparatide has anabolic effects on skeleton.^14,15^ PTH increases the recruitment of mesenchymal cells and their differentiation. Receptors for PTH are found in preosteoblasts, osteoblasts, lining cells, and osteocytes; PTH directly acts on osteoblasts to promote osteoblastogenesis, reduce osteoblast apoptosis, and reactivates quiescent lining cells, leading to an increase in overall osteoblast number and net bone formation.^16–18^ Angiogenesis of bone is a critical event in the process of osteogenesis and bone remodeling. Osteoblasts possess PTH receptors; consequently, VEGF gene expression, protein production, and vascularization are increased *via* the stimulation of PTH.^19,20^ In recent years, the anabolic effects of PTH have been demonstrated by clinical administration: long-term daily subcutaneous injection is the most common strategy; however, this is cumbersome and associated with various side effects.^21^ Several researchers have attempted to extend the application of PTH from systemic treatment to localized applications.^22^ However, systems capable of achieving successful delivery of PTH to local sites to preserve PTH bioactivity and to induce optimal anabolic action are still lacking.

Because PTH is a complicated molecule that has both anabolic and catabolic effects, direct use of the parent peptide at a local site may cause net undesirable catabolic effects.^10^ Recently, we designed and synthesized a new PTH-related peptide (S[PO_4_] VSEI–QLMHN–LGKHL–NSMER–VEWLR–KKLQD–VHNF DDD), PTHrP-1, a repetitive Asp (aspartic acid) sequence, and phosphorylated Ser (serine). Previous studies have shown that calcium and apatite have high affinity for repeating amino acid sequences, such as aspartic acid and glutamic acid.^23,24^ In our previous studies, we used the same process to successfully modify BMP-2 increase its affinity for the scaffold surface to form apatite, and its effects on cell adhesion, proliferation, and differentiation were significantly improved.^25^ To increase the bone-regeneration ability of PTH(1–34) and exert net anabolic effects, we previously applied the same modification was to PTH(1–34) and demonstrated a dose-dependent effect on osteogenic induction.^26^ This modified peptide selectively maintained its anabolic bioactivity and significantly enhanced osteogenic differentiation *in vitro*. Mesoporous bioactive glass (MBG), which has served as a synthetic bone substitute in orthopedics for many years, possesses the ability of bone formation, biodegradation, and drug delivery owing to its high specific surface area, large pore volume and mesoporous structure.^27–29^ In the present study, we used 3D MBG scaffold as a delivery vehicle for this novel peptide to achieve local application; further, we postulate that such a system would improve fusion rate in a rat posterolateral spinal fusion model. Unlike current approaches in tissue engineering, rat bone marrow mesenchymal stem cells (BMSCs) or other cells were not be co-delivered using the present system, as PTH can recruit endogenous mesenchymal cells.^18,30^

## 2. Materials and methods

### 2.1. Preparation of PTHrP-1-MBG scaffolds

A previously reported modified sol–gel and PU foam templating process was used to prepare MBG scaffolds.^31^ Briefly, 4.0 g of F127, 5.2 g of tetraethyl orthosilicate (TEOS), 0.76 g of Ca(NO_3_)_2_·4H_2_O, 0.23 g of triethyl phosphate (TEP), and 1.0 mL of HCl (1 M) were dissolved in 50 g of ethanol and stirred at 40 °C for 1 day, followed by rotary evaporation under vacuum condition for 30 min at 60 °C to obtain an MBG sol with a viscosity of 5 × 10^4^ Pa s. Then, MBG particles as a homogeneous reinforcing agent were added into the sol to form uniform mixture slurry. Next, polyurethane (PU) sponge with desired shape was completely squeezed into the slurry, until the slurry was uniformly coated on the struts. The products were dried at 60 °C for 72 h and calcinated at 600 °C for 6 h in air to obtain the final MBG scaffolds. Then, we adopted a “saturated volume adsorption” strategy for PTHrP-1 immobilization on the scaffold, as previously described.^32^

### 2.2. Characterization of synthesized scaffolds

Brunauer–Emmett–Teller (BET) and Barrett–Joyner–Halenda (BJH) methods were used to determine the surface area, pore size distribution, and pore volume. The porosity of the MBG scaffolds was measured using Archimedes' principle, according to a previous study.^33^ The compressive strength of MBG scaffolds with a size of 10 mm × 5 mm (diameter × height) was tested using a Zwick static materials testing machine (5 kN), at a crosshead speed of 0.5 mm min^−1^.

### 2.3. *In vitro* PTHrP-1 release kinetics

The prepared 0.5 mg PTHrP-MBG scaffolds and 1.0 mg PTHrP-1-MBG scaffolds were respectively incubated in 1 mL of PBS (pH 7.4) at 37 °C for 7 days. The elute of PTHrP-1 peptide was examined 2, 6, 12, and 24 h after incubation, and then examined every other day. At each time point, the supernatant was withdrawn completely, and 1 mL of fresh phosphate-buffered saline (PBS) was added. The amounts of PTHrP-1 peptide in the collected supernatants were measured using the PTH ELISA Kit (Immutopics, Inc., USA).

### 2.4. *In vitro* cellular responses of rat BMSCs (rBMSCs) to PTHrP-1-MBG scaffolds

#### 2.4.1. Cell attachment and proliferation

To assess cell attachment on MBG and PTHrP-1-MBG scaffolds, 1 × 10^5^ rBMSCs (Institute of Biochemistry and Cell Biology, Shanghai, China) were seeded on each scaffold in a 24-well plate and allowed to adhere to the scaffold for 3 h. Subsequently, the cells were incubated in alpha minimum essential medium (α-MEM; Gibco, Invitrogen, Australia) supplemented with 10% fetal calf serum (FCS; Invitrogen) under humidified culture conditions. After 7 days, the samples were removed from the culture wells, rinsed with PBS, and fixed with 2.5% glutaraldehyde in PBS for 1 h. The fixative was removed by washing with buffer containing 4% (w/v) sucrose in PBS and post-fixed in 1% osmium tetroxide in PBS, followed by sequential dehydration in graded ethanol (50%, 70%, 90%, 95%, 100%) and hexamethyldisilizane. The specimens were coated with gold and the morphological characteristics of the attached cells were observed using SEM (FEI Quanta 450). For further investigation of morphology and spreading, a seeding density of 1 × 10^5^ cells per well was applied. After 24 h incubation, samples were fixed with glutaraldehyde solution (2.5%) for 15 min. Then, fixed cells were incubated with FITC-phalloidin (5 g mL^−1^) and DAPI (5 g mL^−1^) for cytoskeleton and cellular nuclei staining, respectively. Cell morphologies were visualized at a magnification of 40× using a confocal laser-scanning microscope (CLSM; Nikon A1R, Japan).

The proliferation of rBMSCs cultured on three groups was determined using the Cell Counting Kit-8 assay (CCK-8; Dojindo Molecular Technologies Inc., Japan). Briefly, rBMSCs were cultured on scaffolds at an initial density of 10^4^ cells per scaffold for 1, 3, and 7 days. Subsequently, 360 μL of culture medium and 40 μL of CCK-8 solution were added to each well at each time point and incubated at 37 °C for another 4 h. An aliquot of 100 μL was taken from each well and transferred to a fresh 96-well plate. The light absorbance of these samples was measured at 450 nm with a microplate reader (Bio-Rad 680, USA). All results were shown as the optical density (OD) values minus the absorbance of blank wells.

#### 2.4.2. Alkaline phosphatase (ALP) activity assay

To assess ALP activity of rBMSCs grown on MBG and PTHrP-1-MBG scaffolds, 1 × 10^5^ rBMSCs were seeded on each scaffold and cultured in a 24-well plate for 7 and 14 days. At the predetermined time point, culture medium was decanted and the cell layer washed gently three times with PBS followed by washing once in cold 50 mM Tris buffer; then, cells were lysed in 200 μL of 0.2% Triton X-100. Lysates were sonicated after being centrifuged at 14 000 rpm for 15 min at 4 °C. Next, 50 μL of supernatant was mixed with 150 μL of working solution according to the manufacturer's protocol (Beyotime, PRC). The conversion of *p*-nitrophenylphosphate into *p*-nitrophenol in the presence of ALP was determined by measuring the absorbance at 405 nm with a microplate reader (Bio-Rad 680). ALP activity was calculated from a standard curve after normalizing to the total protein content, which was determined using a Micro BCA Protein Assay Kit (Pierce), at 570 nm, with a microplate reader (Bio-Rad 680). The results were expressed in μM of *p*-nitrophenol produced per min per mg of protein.

#### 2.4.3. Osteogenesis- and angiogenesis-related gene expression

The expression levels of osteogenic- and angiogenic-related genes (alkaline phosphatase [ALP], bone morphogenetic protein-2 [BMP-2] and bone morphogenetic protein-4 [BMP-4], collagen type I [COL-1], runt-derived transcription factor [RUNX2], osteocalcin [OCN], and vascular endothelial growth factor A [VEGFa]) were measured using quantitative reverse-transcription polymerase chain reaction (qRT-PCR) analysis. Typically, the cells were seeded at a density of 2 × 10^4^ cells per scaffold, cultured for 2 weeks, and harvested at 7 days and 14 days, respectively, using TRIzol Reagent (Invitrogen) to extract RNA. The obtained RNA was reverse-transcribed into complementary DNA (cDNA) using Revert Aid First Strand cDNA Synthesis Kit (Thermo Fisher Scientific, USA) and the qRT-PCR analysis was performed on an ABI Prism 7300 Thermal Cycler (Applied Biosystems, Australia) using SYBR Green detection reagent. The relative expression of the genes of interest was normalized against that of the housekeeping gene β-actin. All samples were assayed in triplicate and independent experiments were performed. The mean cycle threshold (Ct) value of each target gene was normalized against the Ct value of β-actin. The relative expression was calculated using the following formula: 2^−(normalized average Ct)^ × 100.

### 2.5. Western blot analysis for *in vitro* studies

Lysates for western blot analysis were prepared from scaffolds at 7 and 14 days of culture using Phosphosafe lysis buffer (Novagen, USA). Equal amounts of protein lysates were subjected to 4–20% (Bio-Rad) SDS-PAGE. Western blot analysis was carried out with antibodies against phosphorylated-Smad1/5/8 (P-Smad1/5), total Smad1/5/8, phosphorylated-Smad2/3 (P-Smad2/3), total Smad2/3, phosphorylated-p38 (P-p38), total p38, phosphorylated-GSK3β (P-GSK3β), total GSK3β, phosphorylated β-catenin (P-β-catenin), and β-actin followed by 1 : 4000 dilutions of horseradish peroxidase-conjugated IgG antibodies (Bio-Rad) and an enhanced chemiluminescent substrate (Thermo Scientific, Rockford, IL). For detection of P-Smad1/5/8 and total Smad1/5/8, 40 mg of lysate was loaded per lane. For detection P-p38, total p38, P-Smad2/3, total Smad2/3, P-GSK3β, total GSK3β, P-β-catenin, 50 mg of lysate was loaded per lane. All primary phospho antibodies were obtained from Cell Signaling Technology (Beverly, MA), and all primary full-length antibodies were obtained from Santa Cruz Biotechnology (Santa Cruz, CA). Images were analyzed using ImageJ (NIH, Bethesda, MD).

### 2.6. *In vivo* study of a rodent posterolateral spinal fusion model

#### 2.6.1. Surgical procedure and treatment

All experimental rats were bred at the Laboratory Animal Center of Sixth People's Hospital, Shanghai Jiao Tong University School of Medicine. All animal experimental procedures were approved by the Animal Research Committee of Sixth People's Hospital, Shanghai Jiao Tong University School of Medicine. And all animal experiments were performed according to the regulations and guidelines of the animal ethics committee of Shanghai Jiao Tong University School of Medicine. As previously reported,^34^ all surgical procedures were performed on 12 week-old male Sprague–Dawley rats. A posterior midline incision was made over the lumbar spinous processes, after which two separate fascial incisions were made 4 mm from the midline. The L4 and L5 transverse processes were exposed with a muscle-splitting approach *via* sharp dissection using a scalpel blade. After adequate exposure, the fusion bed was irrigated with gentamicin/saline solution, and a high-speed burr was used to decorticate the superficial cortical layer of the transverse processes, followed by implantation of the graft materials at the L4–L5 transverse processes. Eighteen rats were bred and randomly allocated into the following graft study groups: (1) MBG (*n* = 6), (2) 0.5 mg PTHrP-1-MBG (*n* = 6), (3) 0.1 mg PTHrP-1-MBG (*n* = 6). Each animal received an intramuscular injection of antibiotics post-surgically. Following the operation, the animals were allowed free access to food and water and monitored daily for potential complications or abnormal behavior.

#### 2.6.2. Sequential fluorescent labeling

A polychrome sequential fluorescent labeling method was performed on rats sacrificed at week 8 to observe the rate of new bone formation and mineralization. At 2, 4, and 6 weeks after surgery, animals were subjected to an intraperitoneal injection of fluorochromes under ether anesthesia as follows: 30 mg kg^−1^ alizarin red (AL; Sigma) was first injected at 2 week, 25 mg kg^−1^ tetracycline (TE; Sigma, USA) at 4 week, and 20 mg kg^−1^ calcein (CA; Sigma) at 6 week.

#### 2.6.3. Micro-CT

After harvesting the spine at 12 weeks post-operatively, micro-CT (Skyscan 1176, Kontich, Belgium), scanned at 18 μm resolution, of undecalcified samples was used to evaluate new bone formation and fusion rate. Then, 3-D images were reconstructed using the 3-D Creator software. Furthermore, bone volume to total bone volume (BV/TV) and local bone mineral densities (BMDs) were determined using the analysis software.

#### 2.6.4. Manual palpation

After harvesting, the lumbar spines were palpated manually at the level of attempted fusion by two blinded observers, as reported previously.^35^ The observers also palpated the superior and inferior adjacent motion segments. Each motion segment was considered fused and marked as 1 only if there was no motion present in all six directions (flexion and extension, left- and right-side bending, and axial rotation); otherwise, the segment was graded as not fused and marked as 0.

#### 2.6.5. Histological observation

After dehydration in ascending concentrations of alcohol, from 75% to 100%, the undecalcified specimens were embedded in polymethylmethacrylate and 150 mm-thick sections in the orientation of the axial surface were obtained using a microtome (Leica, Hamburg, Germany). The sections were then polished to a final thickness of 40 μm and stained with Van Gieson's picrofuchsin to identify new bone formation and fusion at the interface. Red indicated new bone formation, and the scaffolds were observed as black.

### 2.7. Statistical analysis

The data were collected from three separate experiments and expressed as means ± standard deviation. The one-way analysis of variance and Student–Newman–Keuls *post hoc* tests were used to determine the level of significance, and *P* values less than 0.05 were considered to represent significance.

## 3. Results and discussion

### 3.1. Characterization of synthesized scaffolds

The BET surface areas of the MBG and PTHrP-1-MBG samples were 312.7 ± 19 and 307.3 ± 14 m^2^ g^−1^, respectively. The single-point adsorption total volumes at *P*/*P*_0_ = 0.97 for the MBG and PTHrP-1-MBG samples were 0.332 and 0.341 cm^3^ g^−1^, respectively, and the average mesopore sizes for MBG and PTHrP-1-MBG samples were all 8 nm. The porosities of MBG and PTHrP-1-MBG scaffolds were estimated at 70.3 ± 1.5% and 72.8 ± 1.3%, respectively (Table 1). The compressive strength of MBG and PTHrP-1-MBG scaffolds were 10.3 ± 1.8 MPa and 9.8 ± 2.3 MPa, respectively (Table 1).

**Table 1:** Structural parameters, porosity, and compressive strength of MBG and PTHrP-1-MBG scaffolds

Sample	*S*_BET_ (m^2^ g^−1^)	*V*_p_ (cm^3^ g^−1^)	*D*_p_ (nm)	Porosity (%)	Compressive strength (MPa)
MBG	312.7 ± 19	0.332	8.0	70.3 ± 1.5	10.3 ± 1.8
PTHrP-MBG	307.3 ± 14	0.341	8.0	72.8 ± 1.3	9.8 ± 2.3

MBG scaffolds have been successfully fabricated using a modified sol–gel and PU foam templating process. Using this method, the strut and the pore size, as well as pore morphology, of the scaffolds may be concisely controlled.^31^ In the present study, MBG and PTHrP-1-MBG scaffolds were synthesized with a regular and uniform square macropore structure, and the pore size and porosity were 8 nm and 70%, respectively. The MBG scaffold with a medium porosity of 60–70% possesses the same compressive strength as human trabecular bone.^36^ As the pore size and porosity MBG scaffold we synthesized in this study were 8 nm and 70%, we postulated that the present scaffold provides reliable mechanical strength and a suitable structure for the ingrowth of new bone and blood vessels. The incorporation of PTHrP-1 did not change the original physical characteristics of the MBG scaffold. Such a macroporous structure is essential for cell attachment, migration, flow transport of nutrients, and bone, as well as for blood vessel ingrowth into scaffolds.

### 3.2. *In vitro* release of PTHrP-1

**Fig. 1:**
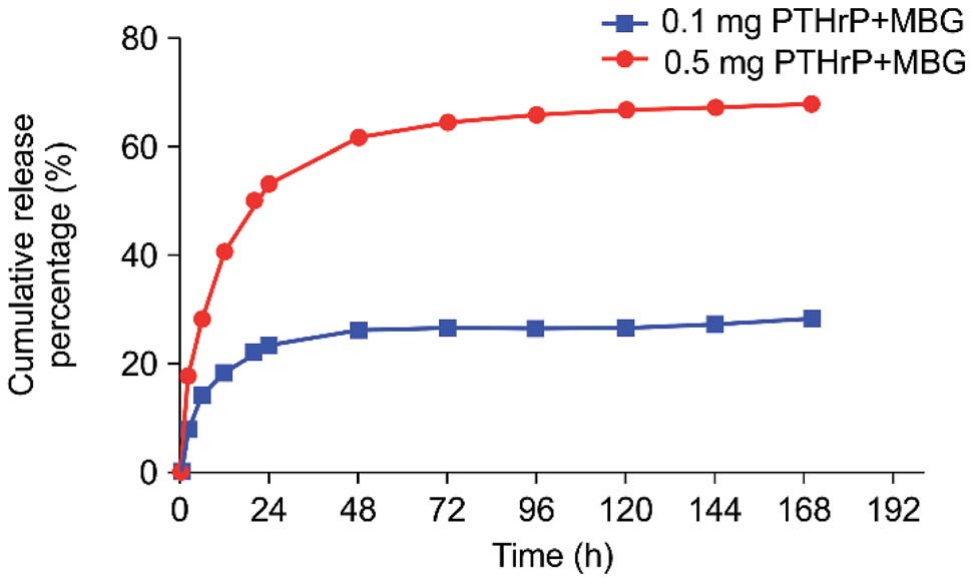
*In vitro* 7 day PTHrP-1 accumulative release curves for the two different-concentration groups.

The effect of PTH on bone was found to be dependent on the delivery pattern; usually, the anabolic actions of PTH are achieved through an intermittent or a short-term, high-dose delivery.^15^ Therefore, the appropriate release pattern of PTH was necessary for bone regeneration. Dang M. *et al.* successfully repaired calvarial bone-defect by locally delivering PTH in a preprogrammed pulsatile manner.^37^ In this study, we postulated that this new peptide could selectively conserve the anabolic part of PTH, thus potentially promoting restricted desirable increases in bone formation with minor, or without, resorption effect. Accordingly, instead of pulsatile delivery, we adopted a continuous delivery method. The *in vitro* PTHrP-1 release profiles were determined using the PTH ELISA Kit; the data showed that around 68% of the total PTHrP-1 was released in the first 60 h for the 0.5 mg PTHrP-1-MBG scaffold group. However, the corresponding value was only 28% for the 0.1 mg PTHrP-1-MBG scaffold group. After 60 h, no significant further release was observed for either group. Overall, rapid continuous release was demonstrated over the duration of observation (Fig. 1). According to our data, MBG scaffolds serve as not only a 3-dimensional porous structure for bone ingrowth, but also an effective delivery vehicle for the novel PTHrP-1. The results also showed that the higher the concentration of PTHrP-1 on the scaffold, the greater the proportion of PTHrP-1 in the scaffolds released. This indicate that high concentrations elicit better release efficiency than low concentrations. Previous research has shown that repeating amino acid sequences, such as aspartic acid and glutamic acid, can bind to calcium and apatite.^23,24^ Release curves for PTHrP-1 also demonstrated the modification endowed PTH(1–34) with high affinity for the scaffold surface. The peptide was not immediately released, but release instead occurred in a more gradual manner, and the release rate gradually decreased over time. Such a release pattern would enable PTHrP-1 to interact with local tissues and cells for a relative long time. Additionally, as the modified molecule has high affinity for apatite, unreleased PTHrP can facilitate newly formed apatite bonds to the scaffold.

### 3.3. *In vitro* bioactivity of PTHrP-1-MBG scaffolds

#### 3.3.1. Cellular responses to PTHrP-1-MBG scaffolds

**Fig. 2:**
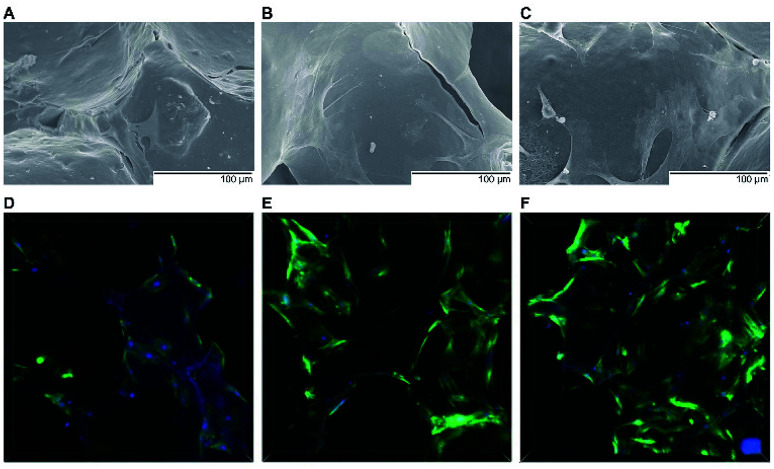
Scanning electron and confocal laser micrographs. SEM images of the attachment of rBMSCs on MBG (A) and 0.1 mg PTHrP-1-MBG (B) and 0.5 mg PTHrP-1-MBG (C) scaffolds after culturing for 7 days; cytoskeletal staining images for MBG (D) and 0.1 mg PTHrP-1-MBG (E) and 0.5 mg PTHrP-1-MBG (F) scaffolds by confocal laser scanning microscopy (green: F-actin, blue: nuclei).

**Fig. 3:**
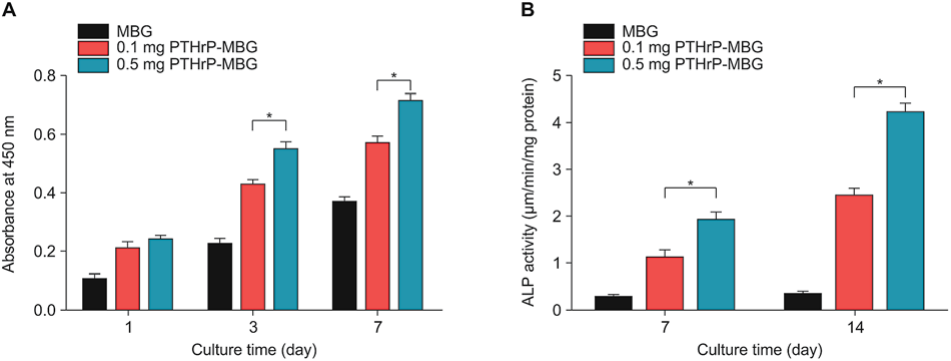
Proliferation (A) and ALP activity (B) of rBMSCs cultured on MBG and PTHrP-1-MBG scaffolds.

**Fig. 4:**
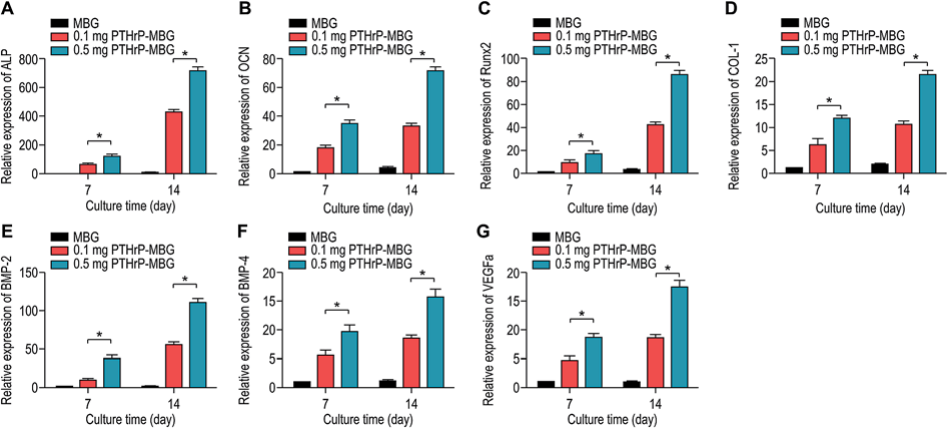
Osteogenic and angiogenic gene expression (ALP (A), OCN (B), RUNX2 (C), Col-1 (D), BMP2 (E), BMP4 (F), VEGFa (G)) of rBMSCs cultured on MBG and PTHrP-1-MBG scaffolds.

To investigate the cell response to PTHrP-1-MBG scaffolds, rBMSCs were used in this study. The attachment and morphology of rBMSCs on MBG and PTHrP-1-MBG scaffolds were observed by SEM (Fig. 2A–C). After 7 days of culture, rBMSCs were attached to the surface of the pore struts, presenting well spread morphology on each type of scaffold. Cytoplasmic webbing, cytoplasmic surface extensions, and flattening of the central mass were most obvious in the 0.5 mg PTHrP-1-MBG group. Compared with the MBG scaffold group, good spreading and a significant increase in the number of cell attachments could be observed in PTHrP-1-MBG scaffold group through confocal laser-scanning microscopy (Fig. 2D–F). These data demonstrate that PTHrP-1 endowed MBG scaffolds with better cytocompatibility and cell adhesive properties. The proliferation of rBMSCs cultured on the MBG and PTHrP-1-MBG scaffolds for 1, 3, and 7 days are shown in (Fig. 3A). As determined by CCK-8 proliferation assay, both MBG and PTHrP-1-MBG scaffolds supported RBMSC proliferation. However, the proliferation rates on the MBG with 0.5 mg PTHrP-1 scaffolds were significantly higher than in the other groups at days 3 and 7 (*P* < 0.05). ALP activity of rBMSCs cultured on MBG and PTHrP-1-MBG scaffolds for 7 and 14 days are shown in (Fig. 3B). Similar to the cell proliferation results, the PTHrP-1-MBG scaffolds exhibited a significant enhanced ALP activity compared WITH the MBG scaffolds, and that of the 0.5 mg was better than in the 0.1 mg group. Cell differentiation of rBMSCs on MBG and PTHrP-1-MBG scaffolds was further evaluated by osteogenic expression, as determined by the expression of osteogenic markers such as ALP, BMP-2, BMP-4, OCN, RUNX2, and CoL-1, as well as that of angiogenic marker VEGFa at 7 and 14 days (Fig. 4). The osteogenic- and angiogenic-related gene expression of rBMSCs was upregulated on MBG with 0.5 mg PTHrP-1 scaffolds compared with that in the other groups, indicating that the addition of PTHrP-1 to the MBG scaffolds promotes osteogenic differentiation and improves angiogenesis.

A significant increase in the number of cells and better attachment profile with increased PTHrP-1 concentration was observed, demonstrating that PTHrP-1 endowed MBG scaffolds with better cytocompatibility and cell adhesive properties. This phenomenon may be attributed to the Wnt pathway, which is considered to be activated by PTH and PTHrP.^38^ ECM plays an important role in cell attachment, while Wnt pathway plays a role in the regulation of the ECM assembly, and modulates local adhesion through the involvement of Wnt components.^39^ For the interaction between PTH and BMSC, the mechanisms involved are multiple; the intrinsic mechanism of such cell attachment and proliferation promotion by PTHrP-1 requires further in-depth study. It is known that MBG possesses osteoinductivity due to abundant calcium and phosphate ion dissolution. ALP activity and osteogenic gene expression of rBMSCs grown on MBG scaffolds and PTHrP-1-MBG scaffolds were assayed to evaluate whether the incorporated PTHrP-1 improves its osteoinductive ability. RT-qPCR results showed that PTHrP-1-MBG scaffolds significantly promoted osteogenic gene expression of ALP, RUNX-2, COL-1, and OCN, as well as that of BMP-2 and BMP-4, indicating that PTHrP-1-MBG scaffolds possess outstanding osteoinductive ability. Angiogenesis is known to be important for bone homeostasis and osteogenesis. Previous studies have shown that osteoblasts possess PTH receptors; consequently, VEGF gene expression and protein production are increased by PTH stimulation.^40^ In this study, expression of VEGFa was also found to be significantly improved by 0.5 mg PTHrP-1 MBG scaffold; however, its effects on angiogenesis remain unknown owing to the lack of verified data on bone angiogenesis *in vivo*. Although the *in vitro* situation does not mimic the architectural and cellular complexity of bone tissues, the positive effects of released PTHrP-1 on the promotion of osteogenesis and angiogenesis were dramatic, and demonstrated a dose-dependent effect. In addition, such dose-dependent effects on osteogenic induction were consistent with our previous study.^26^

Many previous studies have demonstrated the osteoinductive nature of MBG itself; however, our results showed that MBG alone failed to improve rBMSC osteo-differentiation.^40^ Such contradictory results may reflect the fact that most previous studies employed the MC3T3-E1 osteoblast cell for the *in vitro* studies.^41^ Unlike BMSCs, MC3T3-E1 osteoblast cells are a kind of osteoblast precursor cells. Additionally, in previous studies, a special osteoinductive culture medium (containing 10% fetal bovine serum, 50 μg mL^−1^ ascorbic acid, 10 mM β-glycerol-2-phosphate, and 1% penicillin–streptomycin) was used to pre-treat the cells to induce osteoblastic differentiation.^42,43,47^ Because osteoblastic differentiation was initiated before the cells were co-cultured with scaffolds, MBG could promote differentiation. In the present study, rBMSCs were used for the *in vitro* studies, and we did not use any osteoinductive culture medium to pre-treat the cells. MBG could facilitate osteoblastic differentiation after initiation had begun, but its capacity for osteoinduction may not have been strong enough to initiate the osteoblastic BMSC differentiation by itself. Such results further highlight the important role that PTHrP-1 played in the osteoblastic differentiation of rBMSCs.

#### 3.3.2. Western blot analysis

**Fig. 5:**
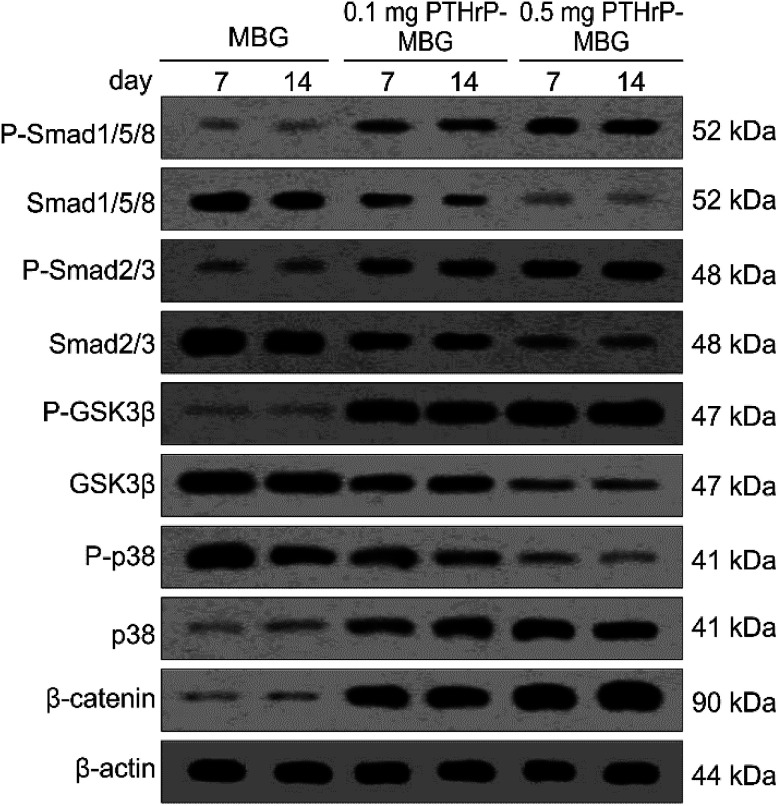
Western blot. Activation of osteogenesis-related signaling pathways by rBMSCs cultured on MBG and PTHrP-1-MBG scaffolds; western blot of phosphorylated Smad1/5/8 (P-Smad1/5/8), total Smad (Smad1/5/8), phosphorylated Smad2/3 (P-Smad2/3), total Smad2/3, phosphorylated-GSK3β (P-GSK3β) and total GSK3β, phosphorylated-p38 (P-p38) and total p38, phosphorylated β-catenin (P-β-catenin) and actin controls.

To elucidate the mechanism underlying the increased osteogenesis observed in PTHrP-1-MBG scaffolds, the canonical (Smad-dependent) and non-canonical (Smad-independent) pathways of BMP receptor signaling and Wnt signaling pathways were investigated (Fig. 5). Both PTHrP-1-MBG scaffold groups demonstrated increased P-Smad1/5/8 and P-Smad2/3 levels; those of the 0.5 mg group were significantly higher than in the 0.1 mg group. Furthermore, P-GSK3β, β-catenin, and p38 dramatically increased in the 0.5 mg PTHrP-1-MBG scaffold group, and were significant higher than that in the other groups. In combination, these data suggest that autogenous activation of the canonical (Smad dependent) BMP receptor signaling pathway in BMSCs can be stimulated by PTHrP-1-MBG scaffolds; such an effect in MBG with 0.5 mg PTHrP-1 scaffolds was significantly higher than in the lower dose group. In addition to the BMP receptor signaling pathway, our data showed that PTHrP-1 scaffolds could also activate Wnt and non-canonical (Smad-independent) pathways of the BMP receptor signaling pathway. MBG scaffolds alone are incapable of stimulating osteo-differentiation of rBMSCs.

PTH exerts both anabolic and catabolic effects when it is systematically used; such dual effects are dependent on its application method.^14^ Intermittent application has been shown to induce the activation of runt-related transcription factor 2 (RUNX2); however, continuous application leads to rapid degradation of RUNX2 and thus reduced bone formation.^44^ Another major effect of intermittent PTH is exerted *via* activation of the canonical Wnt pathway and, in turn, Wnt/β-catenin signaling in osteoblasts. This pathway, which is essential for normal bone formation and cartilage repair, not only mediates the differentiation of stem cells to osteoblasts but also regulates the maturation, proliferation, and anti-apoptosis of osteoblast precursors.^45^ In this study, at the mechanistic level, we demonstrated that the PTHrP-1-MBG scaffold activates BMP signaling pathways through both canonical (Smad-dependent) and non-canonical (Smad-independent) pathways, and it can also activate Wnt signaling pathways. These results indicate that the PTHrP-1 locally delivered through MBG scaffold elicits osteo-differentiation and anabolic actions on rBMSCs. In this study, PTHrP-1 was not released in an intermittent manner; interestingly, it also demonstrated a remarkable anabolic effect. Two explanations may account for such phenomena: one possible reason is that this novel PTHrP-1 could selectively elicit the anabolic effect of PTH, independent of the mode of delivery. Alternatively, PTH may exhibit a different profile when applied locally, as most previous studies were based on the condition that PTH was systematically used. This possibility is consistent with our qRT-PCR showing that MBG alone could not initiate or facilitate the osteoblastic differentiation of rBMSCs. One limitation of this study is that the signaling pathways investigated were only correlated with anabolic activity, while signaling pathways involved in catabolic activity were not monitored. Although the PTHrP-1 on MBG scaffold in our study achieves remarkable activation of bone regeneration, the mechanism underlying increased osteogenesis still need to be elucidated and verified in depth.

### 3.4. Bone regeneration in a rodent posterolateral spinal fusion

#### 3.4.1. Micro-CT measurement

**Fig. 6:**
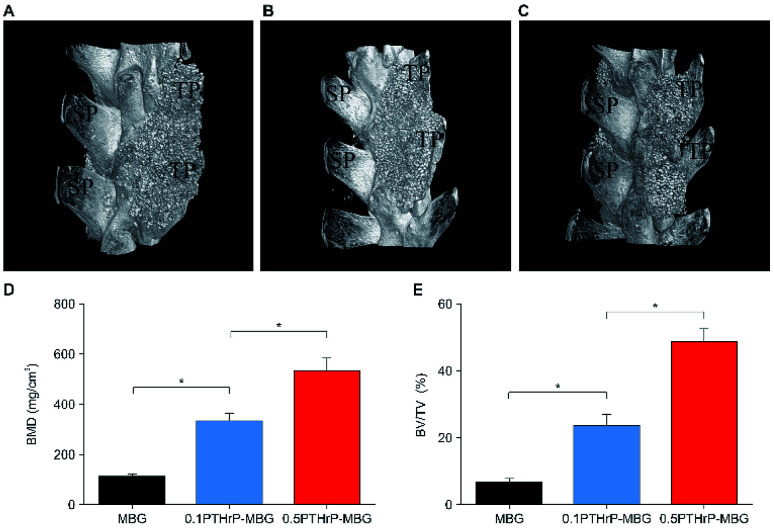
*In vivo* implantation of MBG and PTHrP-1-MBG scaffolds into the rat posterolateral spinal fusion site; micro-CT images show oblique view of the rat spinal column at the implant site 12 weeks post-implantation. Bone formation and complete bridging of two transverse processes was observed through oblique view of CT images in the 0.5 mg PTHrP-1-MBG (A) and 0.1 mg PTHrP-1-MBG (B) scaffold groups, which indicates successful fusion. A clear gap between two transverse processes was observed in MBG group (C), resulting in failure of posterolateral spinal fusion (*n* = 6 per group); SP, spinous process; TP, transverse process. (D) BMD and (E) BV/TV varied in every group (*P < 0.05).

The morphology of the bony bridge between the cranial and caudal transverse processes was reconstructed by micro-CT, and the quantity of the newly formed bone in the implantation sites was calculated by morphometrical analysis. The results showed completed bridging in 0.1 mg PTHrP-1-MBG scaffolds and 0.5 mg PTHrP-1-MBG scaffold groups, while defect bridging was observed in the MBG scaffold group after 12 weeks of implantation (Fig. 6). Rats in the 0.5 mg PTHrP-1-MBG scaffold group formed robust fusion masses that were larger than in all other treatment groups. More importantly, significantly greater new BMD was observed in the 0.5 mg PTHrP-1-MBG scaffold group (535.89 ± 46.50 mg cm^−3^) compared with that in the 0.1 mg PTHrP-1 MBG scaffold group (333.63 ± 28.59 mg cm^−3^) and control group (110.40 ± 12.62 mg cm^−3^). With regard to the morphometric parameter of trabecular bone microarchitecture, the 0.5 mg PTHrP-1-MBG scaffold group exhibited a higher BV/TV ratio (48.81 ± 3.76%) than the 0.1 mg PTHrP-1 MBG scaffold group (23.61 ± 3.24%) and the control group (6.50 ± 1.30%). These results suggest that the PTHrP-1-MBG scaffolds enhanced osteogenesis and achieved satisfactory spinal fusion.

Posterolateral spinal fusion is considered to represent challenging environment for bone regeneration; as a result of load bearing and lack of blood supply, many bone substitutes have failed to achieve successful fusion.^46^ The animal model adopted in the present study represents an efficient way to test the capacity for bone generation by this novel system. As our data showed, both the PTHrP-1-MBG scaffold groups achieved successful fusion in all experimental objects; this was significantly better than in the MBG scaffold group. The MBG scaffold not only serves as a factor delivery vehicle, but also possesses osteogenetic and osteoconductive abilities. Previous studies have demonstrated that MBG itself serves as an outstanding bone substitute in the treatment of critical-size bone defect.^47^ Nevertheless, in this study, satisfactory spinal fusion could not be achieved by MBG alone, suggesting that the incorporated PTHrP-1 has significant beneficial effects on bone generation.

#### 3.4.2. Fluorochrome labeling and histomorphometrical analysis

**Fig. 7:**
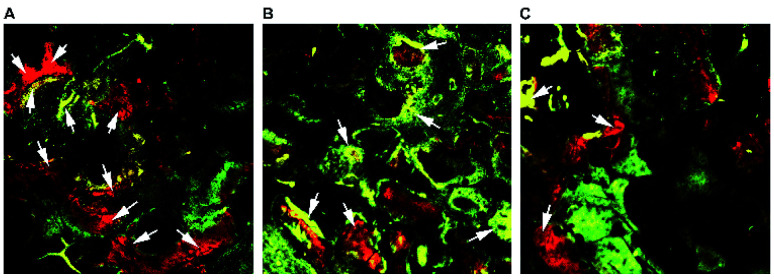
Fluorochrome-labeling analysis. New bone formation and mineralization determined histomorphometrically by fluorochrome-labeling analysis for 0.5 mg PTHrP-1-MBG scaffold group (A), 0.1 mg PTHrP-1-MBG scaffold group (B) and MBG scaffold group (C). As shown by the arrows, higher intensity and a larger continuous area of newly formed bone was seen in the PTHrP-1-MBG scaffold groups. And larger alizarin red labeled area was seen in the 0.5 mg PTHrP-1-MBG scaffold group.

New bone generation and mineralization were determined histomorphometrically by alizarin red, tetracycline, and calcein fluorescence quantification, which represent the mineralization level at 2, 4, 6 week respectively (Fig. 7). The results indicate that PTHrP-1-MBG scaffolds showed larger continuous area of newly formed bone compared with MBG scaffolds (Fig. 7C). The 0.5 mg PTHrP-1-MBG group (Fig. 7A) also showed faster new bone formation than the 0.1 mg PTHrP-1-MBG group and the MBG group (Fig. 7B and C), as its alizarin red labeled area is much larger.

The fluorescent labeling results confirmed that the newly formed bone in all three groups increased with the increase of the implantation time from 2 to 6 weeks. The PTHrP-1-MBG scaffold group showed higher intensity and larger continuous area of newly formed bone than the MBG scaffold group, which means that PTHrP-1-MBG scaffold groups have better bone formation and mineralization. Larger alizarin red labeled area was seen in the 0.5 mg PTHrP-1-MBG scaffold group than other groups, indicating faster new bone formation.

#### 3.4.3. Manual palpation

The 0.5 mg PTHrP-1 MBG scaffold group achieved 100% (6/6) solid fusion, and the 0.1 mg PTHrP-1 MBG scaffold group achieved 83% (5/6) solid fusion. In the MBG group, 1 of 6 rats (16.7%) demonstrated solid fusion. Mean manual palpation scores in the PTHrP-1-MBG scaffold groups were significantly higher than for MBG only.

The characteristics of the MBG scaffold are similar to those of natural bone, as indicated by radiographs; therefore, the quality and solidity of the fusion cannot be evaluated by micro-CT. The manual palpation results showed that PTHrP-1-MBG scaffolds demonstrated superior osteogenic ability compared with the MBG scaffolds. Although the solid fusion rate in the 0.5 mg PTHrP-1-MBG group was statistically higher than that of the 0.1 mg PTHrP-1-MBG group, it could not be concluded that high-dose PTHrP-1 is preferable, owing to the limited number of experimental subjects.

#### 3.4.4. Histological analysis of bone regeneration

**Fig. 8:**
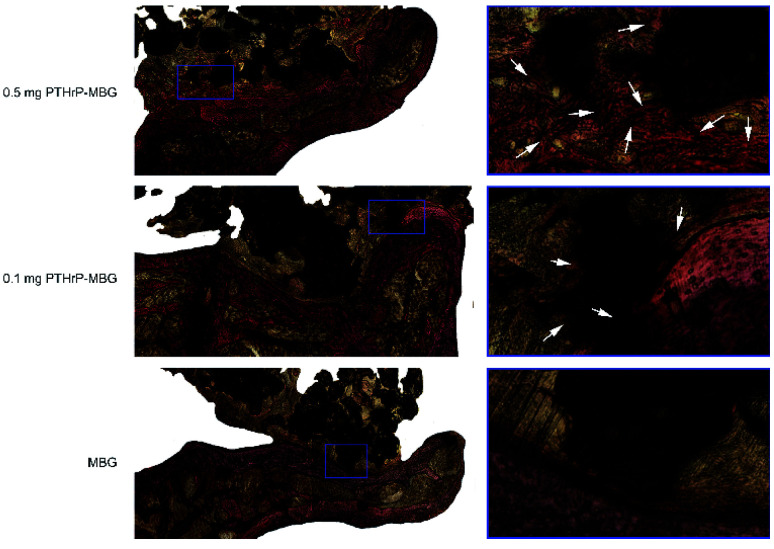
Histological analysis of newly formed bone and fusion at the interface area: the spine was cross-sectionally sliced, and sections were stained with Van Gieson's picrofuchsin. The new bone appears red, and the scaffold appears black. There were significant differences between the MBG control and PTHrP-1-MBG scaffold groups in terms of new bone formation and fusion. For example, newly formed bone occurred at the interface (stained in red and pointed out by the arrows). TP, transverse process.

Undecalcified specimens were stained with Van Gieson's picrofuchsin; the results clearly showed greater newly formed bone and fusion at the interface in the 0.5 mg PTHrP-1-MBG scaffold group compared with that in the 0.1 mg PTHrP-1-MBG scaffolds. Newly formed bone was rarely observed in the MBG group, and a clear boundary could be seen at the interface area (Fig. 8).

The *in vivo* results indicated PTHrP-1-MBG scaffolds possessed good osteogenic and osteoconductive ability to improve novel bone generation and achieve solid spinal fusion. Both the PTHrP-1 and MBG scaffold played an important role in achieving this effect. The MBG scaffold not only served as the PTHrP-1 delivery vehicle but also provided a three-dimensional osteoconductive structure that mimicked natural bone. Many growth factors have been co-delivered with various scaffolds in attempt to facilitate bone regeneration. Among those growth factors, BMP exhibited the best outcome and could improve spinal fusion successfully.^48^ In contrast with PTH, which is believed to be capable of promoting bone remolding, BMP-2 can only induce bone regeneration. It has been reported by many researchers that the use of BMP can result in many side effects, such as hematoma, heterotopic ossification, bone overgrowth, and even tumorigenesis.^49^ Some of these side effects may lead to grave consequences, especially during spinal surgery, considering that hematoma and bone overgrowth occurring at a local site may cause irreversible damage to the spinal cord and nerve roots.^49^ The dual effects of PTH may provide a good solution for addressing these dilemmas. In this study, PTHrP-1 peptide loaded on MBG scaffolds revealed a strong capacity to stimulate new bone formation; in addition, an extremely high fusion rate was achieved by this system. However, because our observation period was only 3 months, the safety profile of PTHrP-1 still needs to be further characterized in the future. In detail, PTHrP-1 released from MBG scaffolds promoted the proliferation, osteogenic differentiation, and angiogenic factor A (VEGFA) expression of rBMSCs. These results suggest that local delivery of PTHrP-1 leads to consistent and robust bone regeneration. Our *in vivo* results demonstrate that enhanced bone regeneration was obtained by local application of this novel PTHrP-1. Additionally, the 0.5 mg PTHrP-1-MBG group exhibited a significantly higher bone formation ability and higher rate of solid spinal fusion than evidenced by the 0.1 mg PTHrP-1-MBG group, suggesting that the effect of PTHrP-1 has a linear relationship with its loading amount. PTH is a complex factor that exerts both catabolic and anabolic effects, depending on a specific temporal profile.^9^ Previous studies have indicated that both N- and C-terminal domains of PTH are critical for activation of relative signaling pathways to fulfill its bioactivity, and modifications of both regions lead to a change in the balance of its dual effect.^50^ Tissue engineering studies aim to develop more effective ways to benefit from the bone regenerative effects of PTH without, or with less, resorption: accordingly, some modified PTHrPs were synthesized as a biased agonist of the PTH receptor.^51^*In vitro* efficacy studies of the effect of various biased peptides on osteoblast proliferation, differentiation, and apoptosis indicate that some of these biased PTH-related peptides represent superior molecules for bone formation.^52^ However, to our knowledge, the present study is the first in which such a biased peptide was tested in an *in vivo* spinal fusion model. The reasons that PTHrP-1-MBG scaffolds elicit such robust bone formation are likely multifactorial; however, this combination approach appears to offer a number of advantages that effectively promote bone regeneration. In general, MBG has high specific surface area, large pore volume, and mesoporous structure; these properties facilitate cell and growth factor attachment and ingrowth of novel generated bone and blood vessels.^53,54^ Therefore, using MBG scaffolds, biomolecules such as PTHrP-1 may be immobilized onto implant surfaces and then locally released to interact with the surrounding tissues for a certain duration to fully activate bone regeneration. Current strategies for skeletal regeneration often require co-delivery of scaffold, growth factors, and cellular material; however, the isolation and expansion of stem cells is time-consuming and expensive.^55^ Importantly, in this study, even though the *in vivo* methods did not involve the co-delivery of progenitor cells, the PTHrP-1-MBG scaffold still showed high-efficiency bone regeneration and good capacity to promote spinal fusion. Studies have identified the pivotal action of PTH and PTHrP on the reactivation of quiescent periosteal lining cells into active osteoblasts and mobilization of the migration of cells from the hematopoietic niche, which leads to an increase in overall osteoblast number and net bone formation.^56^ Therefore, the use of PTHrP-1-MBG scaffold represents an *ex vivo* progenitor cell culture-free implantable strategy for bone regeneration.

Our data demonstrate that the incorporation of MBG scaffolds with local release of PTHrP-1 has a remarkable positive effect on bone regeneration. The present system represents a good candidate for bone graft used in spinal fusion. Nevertheless, the in-depth molecular mechanisms underlying the interaction between released PTHrP-1 and endogenous bone formation-related cells, as well as the optimal loading dose, require further investigation.

## 4. Conclusions

In this study, MBG scaffolds were successfully fabricated by sol–gel and PU foam templating process; then, the novel peptide, PTHrP-1 was immobilized on the scaffolds using the “saturated volume adsorption” technique. The fabricated PTHrP-1-MBG scaffolds afforded ordered mesopores, regular macropores, high porosity, and appropriate mechanical strength; these properties were similar to those of trabecular bone. Importantly, such scaffolds were capable of delivering the PTHrP-1 to the implantation area efficiently. The PTHrP-1-MBG scaffolds stimulated rBMSC adhesion, proliferation, and differentiation, and additionally stimulated angiogenesis simultaneously. The achievement of solid fusion in the rat posterolateral spinal fusion model further showed that the PTHrP-1-MBG scaffolds possess superior osteoinductive activity to enhance bone formation relative to MBG scaffolds. Therefore, the present PTHrP-1-MBG scaffolds represent a promising material for bone regeneration and a good substitute for bone graft, especially in conditions such as spinal fusion.

## Conflicts of interest

The authors declare that they have no competing interests.

## Supplementary Material
